# Plasma CXCL13 but Not B Cell Frequencies in Acute HIV Infection Predicts Emergence of Cross-Neutralizing Antibodies

**DOI:** 10.3389/fimmu.2017.01104

**Published:** 2017-09-08

**Authors:** Jenniffer M. Mabuka, Anne-Sophie Dugast, Daniel M. Muema, Tarylee Reddy, Yathisha Ramlakhan, Zelda Euler, Nasreen Ismail, Amber Moodley, Krista L. Dong, Lynn Morris, Bruce D. Walker, Galit Alter, Thumbi Ndung’u

**Affiliations:** ^1^Africa Health Research Institute, Durban, South Africa; ^2^HIV Pathogenesis Programme, Nelson R. Mandela School of Medicine, Doris Duke Medical Research Institute, University of KwaZulu-Natal, Durban, South Africa; ^3^Ragon Institute of Massachusetts General Hospital, Massachusetts Institute of Technology and Harvard University, Cambridge, MA, United States; ^4^KEMRI-Wellcome Trust Research Programme, Kilifi, Kenya; ^5^Biostatistics Unit, Medical Research Council, Durban, South Africa; ^6^National Institute for Communicable Diseases, Johannesburg, South Africa; ^7^Faculty of Health Sciences, University of the Witwatersrand, Johannesburg, South Africa; ^8^Howard Hughes Medical Institute, Chevy Chase, MD, United States; ^9^Institute for Medical and Engineering Sciences, Massachusetts Institute of Technology, Cambridge, MA, United States; ^10^Max Planck Institute for Infection Biology, Berlin, Germany

**Keywords:** B-cell subsets, acute HIV, CXCL13, cross-neutralizing antibodies, BAFF

## Abstract

Immunological events in acute HIV-1 infection before peak viremia (hyperacute phase) may contribute to the development of broadly cross-neutralizing antibodies. Here, we used pre-infection and acute-infection peripheral blood mononuclear cells and plasma samples from 22 women, including 10 who initiated antiretroviral treatment in Fiebig stages I–V of acute infection to study B cell subsets and B-cell associated cytokines (BAFF and CXCL13) kinetics for up to ~90 days post detection of plasma viremia. Frequencies of B cell subsets were defined by flow cytometry while plasma cytokine levels were measured by ELISA. We observed a rapid but transient increase in exhausted tissue-like memory, activated memory, and plasmablast B cells accompanied by decline in resting memory cells in untreated, but not treated women. B cell subset frequencies in untreated women positively correlated with viral loads but did not predict emergence of cross-neutralizing antibodies measured 12 months post detection of plasma viremia. Plasma BAFF and CXCL13 levels increased only in untreated women, but their levels did not correlate with viral loads. Importantly, early CXCL13 but not BAFF levels predicted the later emergence of detectable cross-neutralizing antibodies at 12 months post detection of plasma viremia. Thus, hyperacute HIV-1 infection is associated with B cell subset changes, which do not predict emergence of cross-neutralizing antibodies. However, plasma CXCL13 levels during hyperacute infection predicted the subsequent emergence of cross-neutralizing antibodies, providing a potential biomarker for the evaluation of vaccines designed to elicit cross-neutralizing activity or for natural infection studies to explore mechanisms underlying development of neutralizing antibodies.

## Introduction

The development of a successful vaccine for HIV-1 will likely require the elicitation of broadly neutralizing antibodies (bNAbs), i.e., antibodies that target fairly conserved epitopes on the HIV envelope spike and, therefore, neutralize the majority of HIV isolates; however, to date, it is not fully understood how such responses can be induced through vaccination. In natural infection, bNAbs only appear after years of infection, developing in a small subset of individuals, although cross-neutralizing antibodies with narrower breadth can be detected earlier and in higher numbers of people (1–8). Thus far, plasma viral load, CD4 count and inflammation have been described as predictors of neutralizing breadth but these would be irrelevant in the context of vaccine trials (1–3, 6, 9). A report investigating bNAb lineages from early infection showed that reverted germline versions bound early autologous envelopes, potentially initiating key B cell selection processes and downstream antibody evolution pathways (10). This observation points to the potential influence of events occurring during hyperacute HIV-1 infection—before peak viremia—on development of cross-neutralizing antibodies, an area that remains unexplored to date.

In primary and chronic untreated HIV-1 infection (PHI and CHI), prior studies, largely cross-sectional in nature, have shown that B cell subset frequencies, defined by surface expression levels of CD21 and CD27, are disrupted (11, 12). Specifically, HIV-1 infected individuals have increased frequencies of immature/transitional B cells, increased tissue-like memory (TLM) B cells with signs of premature exhaustion and decreased frequencies of resting memory (RM) B cells (11–13). Although combination antiretroviral therapy (cART) initiated during chronic infection results in normalization of most B cell subsets, memory B cell defects persist and only show significant recovery if patients initiate treatment early in the course of infection (14–20). It remains unknown whether pre-infection B cell subset frequencies and changes occurring during hyperacute HIV-1 infection (or immediately following encounter with antigen following vaccination) might be used to predict the emergence of early cross-neutralizing antibodies and thus help guide vaccine strategies to drive this activity.

HIV-1 bNAbs generally have unusual features including high levels of somatic hypermutation in both complementarity-determining region (CDR) loops and framework regions, long heavy chain CDR 3 (CDRH3), and a propensity toward autoreactivity (21–24). Indeed, accumulating data now show that levels of the chemokine CXCL13, produced by T follicular helper cells (Tfh), play a key role in the quality of the germinal center (GC) reaction and predict development of cross-neutralizing antibodies in HIV-infected patients (25–27). The B cell-associated cytokine B cell activating factor (BAFF) can also potentially influence the survival and class switching of unique autoreactive B cells likely to generate cross-neutralizing antibodies (28–30). Thus far, BAFF has been shown to augment development of cross-neutralizing antibodies in animal models when used as an adjuvant or supplied exogenously (31, 32) although this was not true in a cohort of subtype B infected individuals (25). Whether the levels of these two key B cell associated cytokines during hyperacute HIV-1 infection can predict subsequent development of cross-neutralizing antibodies later remains to be determined.

We sought to understand the dynamics of the B cell response, with respect to subset changes and B cell associated cytokines, prior to infection, and during hyperacute infection and how they might influence development of cross-neutralizing antibodies. Additionally, the impact of cART initiated during the acute phase of infection on these factors was evaluated. We used pre- and post-HIV-1 subtype C infection samples from young women enrolled in a study termed Females Rising through Education, Support and Health (FRESH) in the KwaZulu-Natal province of South Africa (33). We measured the dynamics of B cell subsets, plasma levels of BAFF and CXCL13 before infection and longitudinally during hyperacute HIV-1 infection and determined their influence on the emergence of cross-neutralizing antibodies at approximately 1 year postinfection (PI). Our data demonstrate that B cell defects reported in PHI and CHI emerge during hyperacute HIV-1 infection in women who do not initiate early treatment and are abrogated with immediate treatment, indicative of the influence of viral load on the observed changes. However, these dramatic B cell changes occurring in hyperacute infection did not predict the emergence of cross-neutralizing antibodies. In contrast, changes in BAFF and CXCL13 during hyperacute infection were not directly associated with viral loads. Importantly, we found higher levels of CXCL13 during hyperacute infection in individuals who subsequently developed detectable cross-neutralizing antibodies within 1 year of infection compared to those who did not. Hence our data from subtype C hyperacute infection confirm the utility of CXCL13 levels early in infection as a biomarker for possible superior GC activity associated with emergence of cross-neutralization antibodies.

## Materials and Methods

### Study Population and Blood Samples

Females Rising through Education, Support and Health is a longitudinal cohort study of 18- to 23-year-old HIV-1-negative women at high risk of HIV-1 infection established in the Umlazi Township of Durban, KwaZulu-Natal, South Africa. Cohort recruitment and follow-up details have been comprehensively described elsewhere (33–35). Briefly, blood samples were obtained at study entry and every 3 months thereafter from HIV-1-negative study participants. Study subjects attended twice-weekly sessions in which trained counselors offered a comprehensive life and job skills, empowerment and HIV-1 prevention curriculum. During the twice-weekly visits, finger prick blood samples were taken for monitoring of plasma HIV-1 RNA, with results available within 24 h. Participants with a positive RNA test were contacted immediately, counseling was provided, and blood samples were collected. Subsequently, longitudinal PI venous blood samples were obtained at regular intervals through peak viremia and beyond. Peripheral blood mononuclear cells (PBMCs) were frozen from each venous blood draw for future analysis. Initially, participants identified with onset of plasma viremia were closely monitored and referred for cART if meeting eligibility according to South African guidelines (36). Beginning July 2014, the study protocol was amended and participants with onset of HIV-1 plasma viremia were initiated on cART immediately using a standard 3-drug regimen of tenofovir disoproxil fumerate 300 mg, emtricitabine 200 mg, and efavirenz 600 mg (TDF/FTC/EFZ). From July 2015, participants with acute viremia received early treatment with TDF/FTC/EFZ plus raltegravir (RAL) 400 mg twice-daily, with RAL withdrawn two months after suppression of plasma viremia to undetectable levels.

### B Cell Phenotyping

Frozen PBMCs were thawed and allowed to rest for 2 h before being used for phenotypic analysis using a panel of fluorescently labeled monoclonal antibodies reactive with the following cell surface markers: BV711 conjugated antihuman CD3 (BioLegend, San Diego, CA, USA), BV450 mouse antihuman CD21 (BioLegend, San Diego, CA, USA), Qdot 605 mouse antihuman CD19 (Life Technologies, Carlsbad, CA, USA), PE mouse antihuman CD27 (BD Biosciences, San Jose, CA, USA), Alexa Fluor 700 mouse antihuman CD38 (BD Biosciences, San Jose, CA, USA), and aqua viability dye (Life Technologies, Carlsbad, CA, USA). Rested PBMCs were stained with 200 µl of diluted viability dye and allowed to incubate in the dark for 15 min at RT. Thereafter, cells were washed twice in phosphate-buffered saline (PBS) and then 100 µl of the cocktail of antibodies was added to 2 × 10^6^ cells and incubated for 15 min at room temperature. Thereafter, tubes were washed with 3 ml PBS and centrifuged at 600 × *g* for 5 min. Supernatant was discarded and 100 µl of 2% paraformaldehyde was added to each tube. Samples were then acquired on the LSRFortessa (Becton Dickinson, Franklin Lakes, NJ, USA) and data analyzed on FlowJo version 9.8.3 (FlowJo LLC, Ashland, OR, USA).

### Determination of Plasma BAFF and CXCL13 Levels

BAFF and CXCL13 levels were determined by ELISA (R&D systems, Minneapolis, MN, USA) using the manufacturer’s protocol. Plasma samples were thawed slowly on ice, spun down and the clear supernatant used immediately for the assays.

#### Neutralization Assays

Neutralization activity was determined using a previously described standard TZM-bl cells based assay (NIH AIDS Research and Reference Reagent Program, Division of AIDS, NIAID, NIH) (37). This assay measures Tat-induced luciferase reporter gene expression after infection by HIV-1 Env-pseudotyped viruses with neutralization quantified by reduction in relative light units in TZM-bl cells in the presence of HIV-1-positive plasma. Samples were used at 1:50 dilution, and the ID50 was calculated as the reciprocal dilution at which 50% of the virus was inhibited.

#### Data Analysis

Non-parametric Spearman’s rank tests were used to test for correlations and a 2-tailed Mann–Whitney test was used to evaluate unpaired groups. Wilcoxon matched signed-rank test was used to evaluate paired samples. To assess the relationship between each B cell subset and time, varying viral load, CD4 count, BAFF, and CXCL13 adjusted for days PI, linear mixed effects models with random (subject specific) intercepts were fitted to the B cell data. Due to the complex non-linear evolution of B cell subsets over time, an unstructured mean was considered. The variables of interest (CD4 counts, viral load, CXCL13, and BAFF levels) were treated as time dependent covariates in the model, separately. B cell subsets (the outcome) were log transformed. By comparison of Akaike information criterion and Bayesian information criterion, the most suitable model was that with a random intercept and residuals which follow an autoregressive (1) structure. *p*-Values less than 0.05 were considered significant. Data analysis was performed in Graphpad Prism version 6 (Graphpad Software, San Diego, CA, USA) and Stata version 13.0 (Statacorp, College Station, TX, USA).

#### Ethics Statement

Study subjects provided written informed consent for participation in the study. Ethical approval was provided by the Biomedical Research Ethics Committee of the University of KwaZulu-Natal and the Institutional Review Board of Massachusetts General Hospital.

## Results

### Rapid but Transient Changes in Frequencies of B Cells and B-Cell Subsets in Acute HIV-1 Subtype C Infection

**Figure 1:**
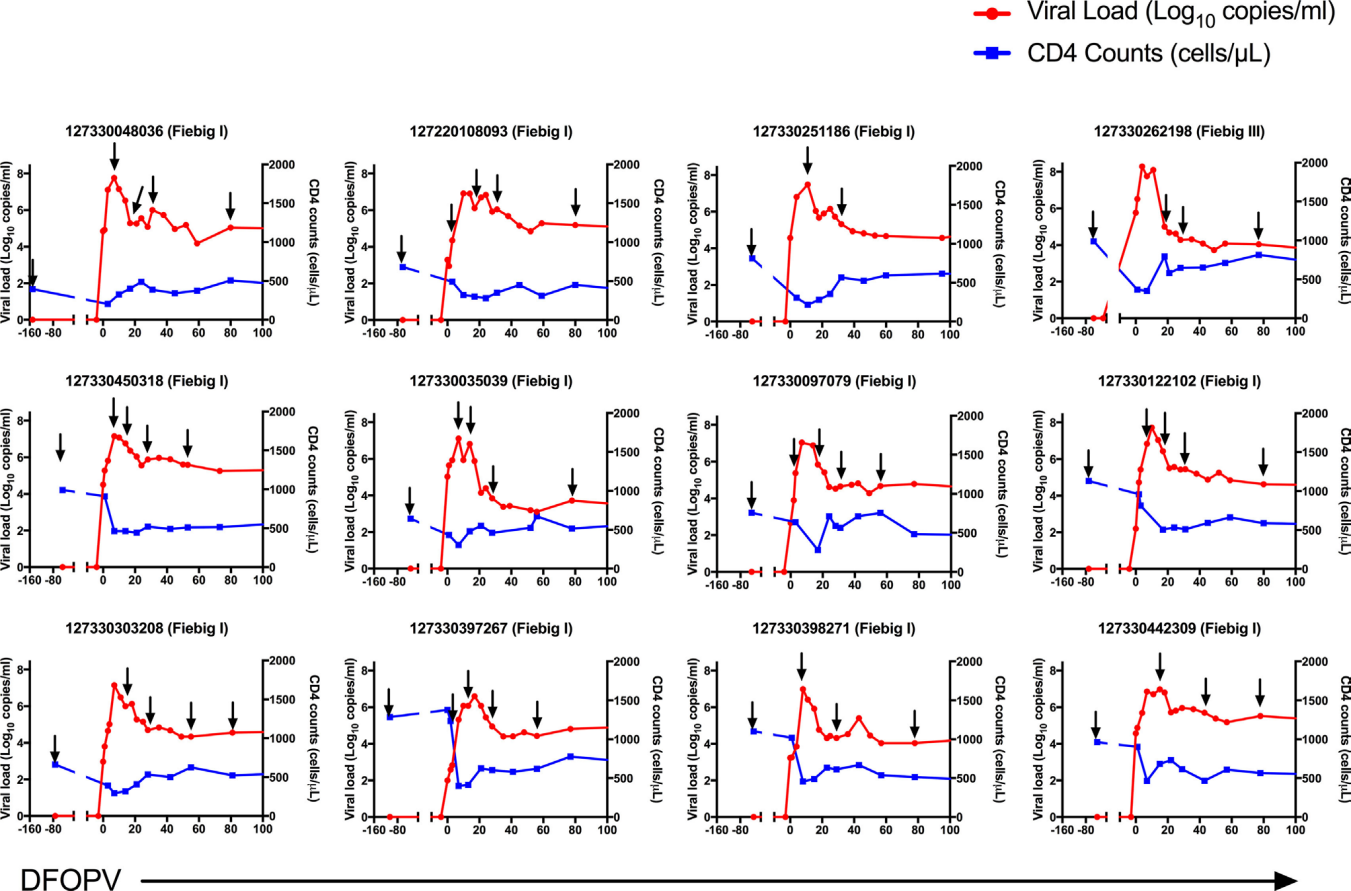
Dynamics of plasma viral loads and CD4 counts during hyperacute HIV-1 subtype C infection in absence of early antiretroviral treatment. Plasma HIV-1 RNA levels (red) and absolute CD4 counts (blue) before HIV infection and following onset of detectable plasma viremia in 12 subjects with hyperacute HIV infection that were not initiated on early antiretroviral treatment. The arrows indicate time-points used for B cell analysis. DFOPV, days following onset of plasma viremia.

**Figure 2:**
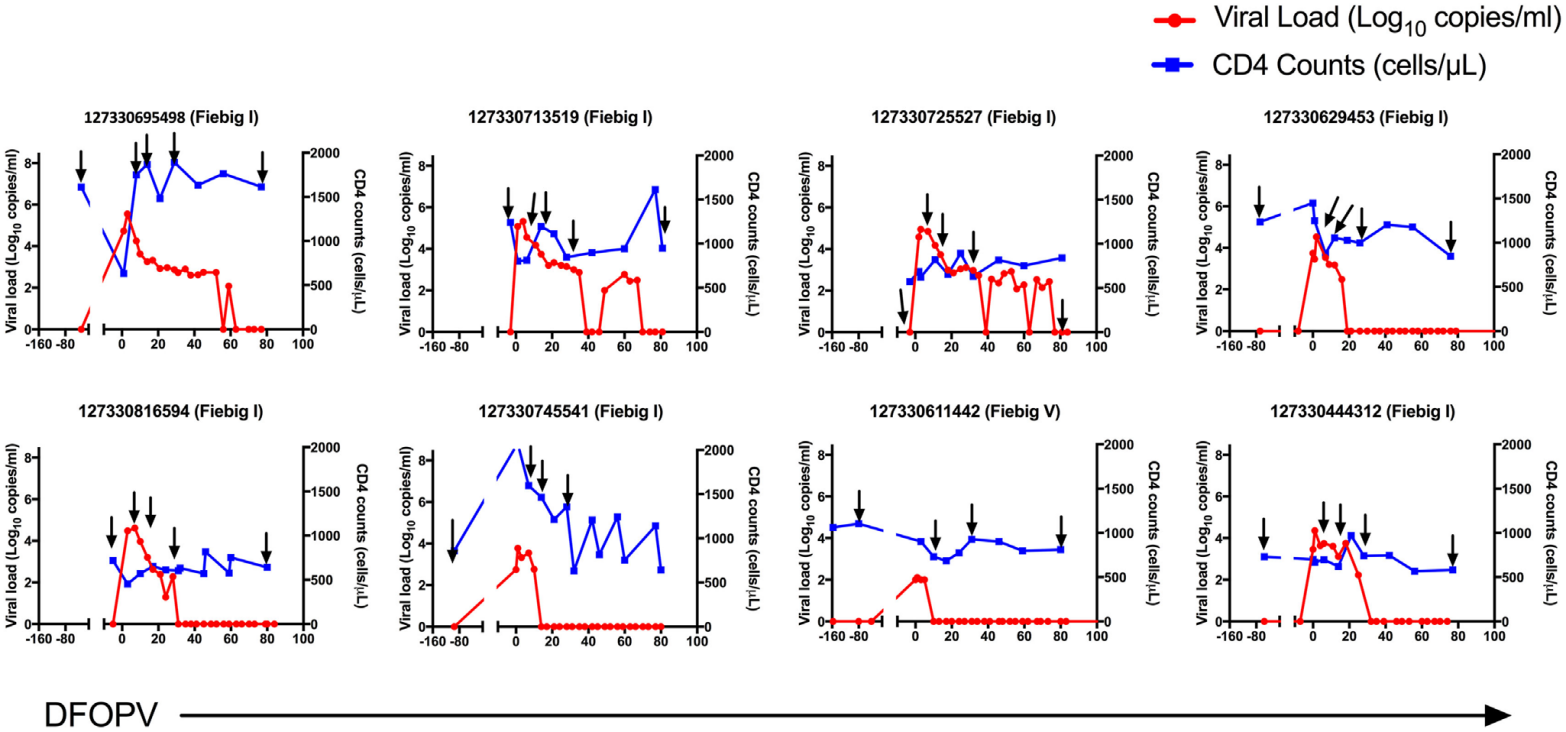
Dynamics of plasma viral loads and CD4 counts during hyperacute HIV-1 subtype C infection with early initiation of antiretroviral treatment. Plasma HIV-1 RNA levels (red) and absolute CD4 counts (blue) before HIV infection and following onset of detectable plasma viremia in eight subjects with hyperacute HIV infection that were initiated on early antiretroviral treatment. The arrows indicate time-points used for B cell analysis. DFOPV, days following onset of plasma viremia.

Pre-infection samples were obtained from all participants in this study. Among the 12 untreated participants, the initial PI samples were obtained in Fiebig stage I for 11 participants and Fiebig stage III for one participant, providing us the opportunity to study very early changes in B-cell subsets and associated cytokines, and to determine how early events might influence the emergence of cross-neutralizing antibodies. Multiple samples were also obtained from participants prior to peak viremia, and during resolution of peak viremia to a viral load set-point. Ten early treated women were also studied, representing a subset of persons within our cohort who initiated standard first line treatment (TDF/FTC/EFZ) within less than 3 days of HIV-1 RNA detection. Among them, the initial PI samples were obtained in Fiebig stage I for 8 participants and Fiebig stage V for two participants. If a participant did not have a sample at 3 months after HIV-1 RNA detection, an alternative sample at 2 months was used (Figures 1 and 2).

**Figure 3:**
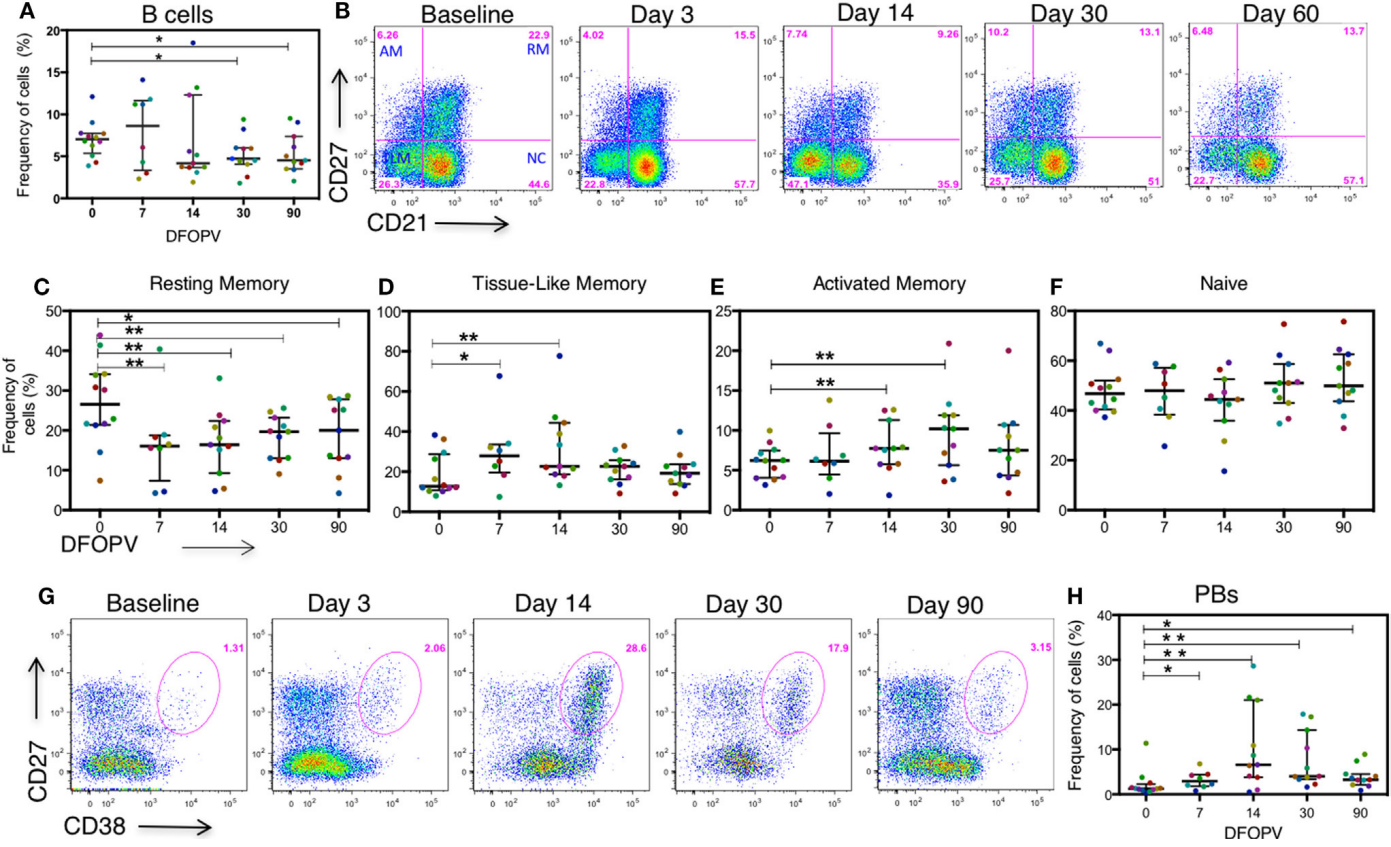
Frequency of B cells before and during acute HIV-1 subtype C infection in absence of early antiretroviral treatment. B cells were defined by the expression of CD19 on CD3^−^ peripheral blood lymphocytes. B cell subsets were defined by the expression of CD27 and CD21 on CD3^−^CD19^+^ lymphocytes. Plasmablasts (PBs) were defined as CD27^+^CD38^+++^ cells on CD3^−^CD19^+^ peripheral lymphocytes. Subsets were analyzed on longitudinal AHI samples obtained in the first ~90 DFOPV and compared to matched baseline values. Panel **(A)** shows a summary of the frequency of B cells as a percentage of lymphocytes overtime. Panel **(B)** is representative data showing B cell subsets from baseline (before infection) to ~60 DFOPV, example from participant 127-033-0097-079. Panels **(C–F)** represent frequencies of B cell subsets; **(C)** resting memory, **(D)** tissue-like memory, **(E)** activated memory, and **(F)** naïve cells. **(G)** Representative data from participant 127-033-0108-093 shows kinetics of PBs from baseline to ~90 DFOPV. **(H)** A comparison between frequencies of PBs at baseline and longitudinal time-points up to ~90 DFOPV. Horizontal lines represent median values and each color represents one patient. DFOPV, days following onset of plasma viremia, and time-point “0” represents baseline (visit prior to infection). *p*-Values were calculated by Wilcoxon matched signed-rank test (*p < 0.05, **p < 0.005).

It has previously been reported that HIV-1 uninfected people have geography- and gender-dependent differences in lymphocyte counts (38–40). We, therefore, first established the baseline (pre-infection) frequency of B cells defined as the percentage of CD3^−^CD19^+^ cells of the total live peripheral blood lymphocyte population in the 12 untreated women. We found that on average these cells accounted for 7% of the peripheral blood lymphocytes at baseline (range 3.9–12.1%), which was lower than what has been observed in geographically different cohorts from Uganda (40). Following infection, three untreated individuals showed a transient increase in frequency of total B cells at days 7 and 14, although these populations decreased thereafter (Figure 3A). Overall, the median frequency of total circulating B cells was significantly lower compared to baseline pre-infection levels at 30 days (*p* = 0.024) and 90 days (0.048) following onset of plasma viremia (DFOPV) (Figure 3A). These data suggest that HIV-1 subtype C infection in an African population alters B cell frequencies presumably through indirect killing or redistribution of B cells, or through expansion of other lymphocyte populations, resulting in decreased proportions of B cells in the periphery over time.

The availability of pre-infection and hyperacute infection samples allowed us to determine baseline frequencies and subsequent kinetics of alterations in B cell subsets with the goal of defining early signatures associated with emergence of cross-neutralizing antibodies. Different clades of HIV-1 differ in pathogenicity and rates of disease progression. Thus, we hypothesized that the B cell kinetics in this clade C cohort might be unique if clade specific features, such as replicative capacity, are a determinant of B cell subset alterations (41–43).

We first determined the kinetics of the four previously described B cell subsets [activated memory (AM), RM, TLM, and naïve cells (11, 12)] defined by the expression of CD21 and CD27 on CD19^+^ mature B cells as shown in representative data (Figure 3B). There was a rapid decrease in the frequencies of RM cells (CD21^+^CD27^+^) noted at 7 DFOPV (medians; 26.55 and 16.5%, range 7–43.9 and 1–21.5% for baseline and 7 DFOPV, respectively), concurrent with an increase in TLM cells (CD21^−^CD27^−^) (medians; 12.7 and 27.85%, range 7.94–38.3 and 7.49–67.7% for baseline and 7 DFOPV, respectively). The frequencies of RM cells remained significantly lower than baseline throughout the time-points tested thereafter in the first 3 months PI (*p* = 0.008, 0.001, 0.005, and 0.019 for 7, 14, 30, and 90 DFOPV, respectively) (Figure 3C). Compared to baseline, frequencies of TLM cells were significantly higher at 7 and 14 DFOPV (*p* = 0.039 and 0.0001, respectively). Thereafter, frequencies of TLM cells remained elevated in most individuals though not statistically significant through to 90 DFOPV (Figure 3D). Importantly, neither RM nor TLM frequencies were restored to baseline values by ~90 DFOPV. We observed a significant expansion of AM cells (CD21^-^CD27^+^) by 14 DFOPV (*p* = 0.005) that persisted at 30 DFOPV (*p* = 0.010) when a peak was reached followed by contraction to near baseline values in some of the individuals by 90 DFOPV (*p* = 0.083) (Figure 3E). No changes were observed in the frequency of naïve B cells (CD21^+^CD27^-^) following HIV-1 infection (Figure 3F).

Plasmablasts (PBs) represent immunoglobulin secreting terminally differentiated B cells, which are transiently enriched in blood during infection or vaccination (44–46). To define PB kinetics in HIV-1 infection, we assessed the frequencies of CD3^−^CD19^+^CD27^+^CD38^+++^ cells before and upon HIV-1 infection. At pre-infection baseline, the median frequency of PBs was 1.26% (range 0.321–11.4%) of the total B cell population. Upon infection, there was a transient expansion of the PB population as shown in the representative example (Figure 3G) that peaked by ~14 days (medians 1.26 and 6.58%, range 0.321–11.4% and 0.532–28.6% for baseline and 14 DFOPV, respectively). Following HIV-1 infection, frequencies of the PB population remained significantly elevated at all time-points tested (*p* = 0.016, 0.002, 0.002, and 0.019 for 7, 14, 30, and 90 DFOPV, respectively) (Figure 3H). Thus, these data illustrate that untreated subtype C acute HIV-1 infection is associated with rapid changes in frequencies of circulating B cell subsets characterized by an increased frequency of AM, TLM, and PBs but a decrease in RM cells.

### Increase in Plasma BAFF and CXCL13 Levels in Acute HIV-1 Subtype C Infection

**Figure 4:**
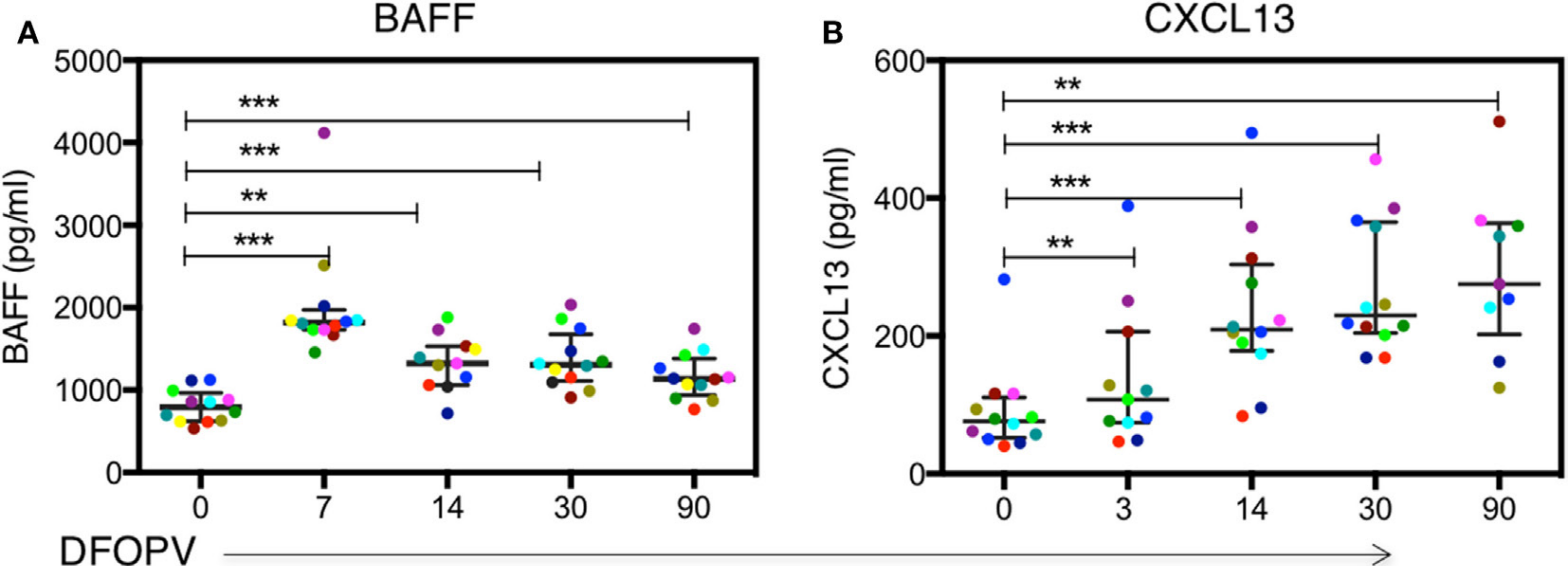
Dynamics of plasma BAFF and CXCL13 levels following acute HIV-1 subtype C in absence of early antiretroviral treatment. Panels **(A,B)** show kinetics of BAFF and CXCL13 levels determined by ELISA using plasma samples obtained longitudinally from untreated patients within the first ~90 DFOPV and compared to matched baseline values. Horizontal lines represent median values and each color represents one patient. DFOPV, days following onset of plasma viremia. *p*-Values were calculated by Wilcoxon matched signed-rank test (**p < 0.005, ***p < 0.0001).

Given the early increase in PBs and alterations in B cell subsets, and considering that acute HIV infection has previously been associated with a cytokine storm that may have profound long-term immunological consequences (47), we next sought to determine whether there were changes following HIV infection in soluble factors associated with B cell activation, survival, and maturation. Specifically, we investigated the levels and kinetics of BAFF, a cytokine important for B cell survival, and CXCL13, a chemokine responsible for B cell trafficking to GCs and potentially responsible for the expansion of PBs (26, 48). The median plasma level of BAFF at baseline was 795 pg/ml (range 536–1,121 pg/ml). These levels increased rapidly and significantly upon infection peaking by 7 DFOPV at a median of 1,817 pg/ml (range 1,457–4,119 pg/ml, *p* = 0.0005) and remained significantly higher throughout the first 90 DFOPV (*p* = 0.005 for 14 DFOPV and *p* = 0.0005 for both 30 and 90 DFOPV) (Figure 4A). The median plasma CXCL13 level at baseline was 76 pg/ml (range 40–282 pg/ml). Similar to BAFF, CXCL13 levels were elevated upon infection although the increase was progressive with the highest median of 275 pg/ml (range 125–511 pg/ml) being registered 90 DFOPV (the last visit analyzed). Compared to baseline, the measurements remained significantly higher throughout the time-points analyzed in the first 90 DFOPV (*p* = 0.003, 0.0005, 0.0005, and 0.0039 for 3, 14, 30, and 90 DFOPV, respectively) (Figure 4B). Thus, acute HIV-1 infection is associated with rapid and gradual increase in plasma levels of B cell-associated cytokines BAFF and CXCL13, respectively.

### Viral Loads Directly Drive Changes in B Cell Subset Frequencies but Not Levels of Plasma BAFF and CXCL13

Viral loads and associated immune activation in chronic infection have been linked to changes in B cell subsets and development of bNAbs during chronic infection (9, 49, 50). To determine whether viral replication was associated with the observed changes, we first assessed the relationship between contemporaneous viral loads, CD4^+^ T cell counts and B cell frequencies over time. We found a negative trend and significant relationship between PBs and CD4 counts at baseline (rho = −0.52, *p* = 0.080) and 7 DFOPV (rho = −0.82, *p* = 0.023), respectively (data not shown). Next, we used linear mixed effect models to investigate the overall relationship between the rapid changes in viral loads, CD4^+^ T cells and observed changes in B cell subset frequencies over time. Viral load was negatively associated with RM cell frequencies (*p* < 0.0001), positively associated with TLM cells (*p* = 0.005) but no significant associations with AM and PBs were observed (Table 1). In contrast, CD4^+^ T cell counts were positively associated with RM cells (*p* = 0.001) and negatively associated with TLM cells (*p* = 0.039) and AM cells (*p* = 0.009) (Table 1). Further, we used a model of a similar form to determine the relationship between changing levels of BAFF, CXCL13, and markers of disease progression. Interestingly, there was no significant relationship between viral loads and BAFF (*p* = 0.511) or CXCL13 (*p* = 0.940). Furthermore, no association was found between CD4 cell counts and BAFF plasma levels; however, we observed a negative association between CD4^+^ T cell counts and CXCL13 plasma levels (*p* < 0.0001) (data not shown). We also found that BAFF levels were significantly associated with high frequencies of AM (*p* = 0.006) and PBs (*p* = 0.026) cells (Table 1). In contrast there was no significant relationship between plasma levels of CXCL13 and any B cell subset frequencies (Table 1). Taken together, these data confirm the direct relationship between viral loads and B cell subset frequencies but not BAFF and CXCL13. We, therefore, show for the first time that accumulation of TLM cells, which has mostly been associated with chronic infection, manifests within days of infection and associates with viral loads. Furthermore, the observation of a positive correlation between BAFF levels and specific B cell subsets (AM and PBs) during hyperacute HIV-1 infection may suggest a direct stimulation and/or maintenance of these subsets by this cytokine.

**Table 1:** Linear mixed effect models for the relationship between viral loads, CD4 counts, CXCL13, and BAFF over time and B cell subsets in absence of early antiretroviral treatment.

		HIV-1 infected untreated				
	B cell subset	Activated memory	Resting memory	Tissue-like memory	*Naive*	Plasmablasts
Viral Loads	Coef (SE)	0.0604 (0.037)	−2.5253 (0.5238)	0.1083 (0.0388)	−0.3031 (1.2403)	0.1384 (0.0973)
	*p*-Value	0.103	**<0.0001**	**0.005**	0.807	0.155
CD4 counts	Coef (SE)	−0.0006 (0.0002)	0.0119 (0.0035)	−0.0005 (0.0002)	0.0028 (0.0074)	−0.0010 (0.0005)
	*p*-Value	**0.009**	**0.001**	**0.039**	0.705	0.078
CXCL13	Coef (SE)	0.0008 (0.0006)	−0.0160 (0.0106)	0.0007 (0.0007)	0.0026 (0.0188)	0.0010 (0.0013)
	*p*-Value	0.207	0.13	0.309	0.889	0.424
BAFF	Coef (SE)	0.0003 (0.0001)	−0.0015 (0.0025)	0.00002 (0.0001)	0.0038 (0.0040)	0.0007 (0.0003)
	*p*-Value	**0.006**	0.553	0.909	0.373	**0.026**

*Significant p values are shown in bold*.

### Early cART Blocked Changes in B Cell Subset Frequencies and Plasma Levels of BAFF while Diminishing Changes in Levels of Plasma CXCL13

**Figure 5:**
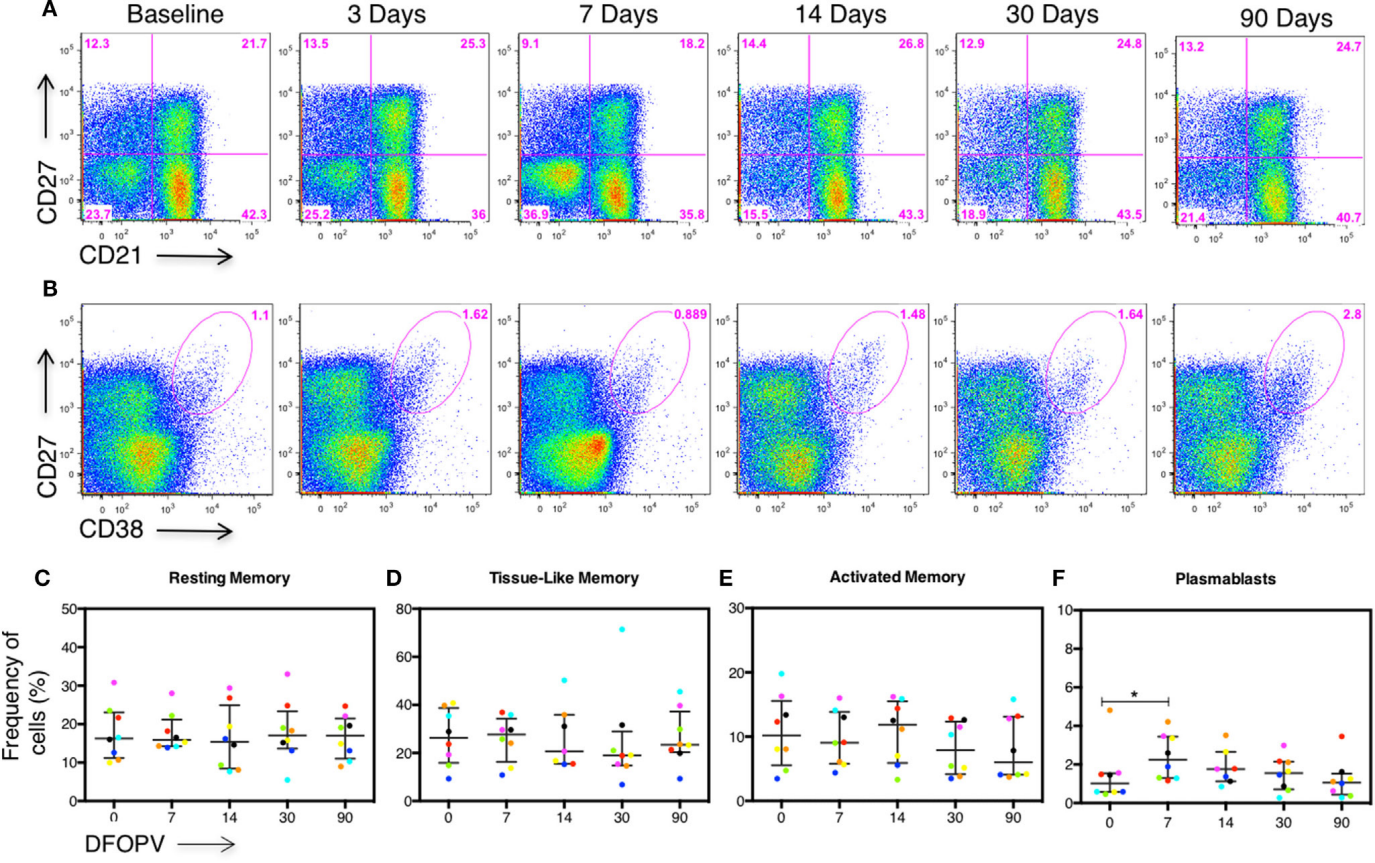
Early combination antiretroviral therapy (cART) blocks B cell subset changes. Peripheral blood mononuclear cells obtained longitudinally from eight individuals who initiated cART during Fiebig stage I–V were used to define B cell subsets and plasmablasts (PBs) by the expression of CD27, CD21, and CD27^+^CD38^+++^ cells on CD3^−^CD19^+^ peripheral lymphocytes, respectively. B cell frequencies from the first ~90 DFOPV were compared to matched baseline values. Panels **(A,B)** are representative data from participant 127-033-0629-453 showing kinetics of B cell subsets and PBs, respectively, from baseline (before infection) to 90 DFOPV. Panels **(C–F)** represent frequencies of B cell subsets; **(C)** resting memory, **(D)** tissue-like memory, **(E)** activated memory, and **(F)** PB cells. Horizontal lines represent median values and each color represents one patient. DFOPV, days following onset of plasma viremia, and day “0” represents baseline (visit prior to infection).

Following our observation that changes in B cell subset frequencies are influenced by viral load, we next determined whether in the absence of persistent antigenemia the levels of the different B cell subsets, as well as B cell associated cytokines BAFF and CXCL13, would remain normal. Remarkably, there were no significant B cell subset changes observed (representative data Figures 5A,B and summary Figures 5C–E) except for an increase in PBs at 7 DFOPV (*p* = 0.039) (Figure 5F) but at lower frequencies than what was observed in untreated women (Figure 3). Indeed, frequencies of AM cells at 30 and 90 DFOPV trended toward being lower than baseline (*p* = 0.109 and 0.078, respectively, data not shown).

**Figure 6:**
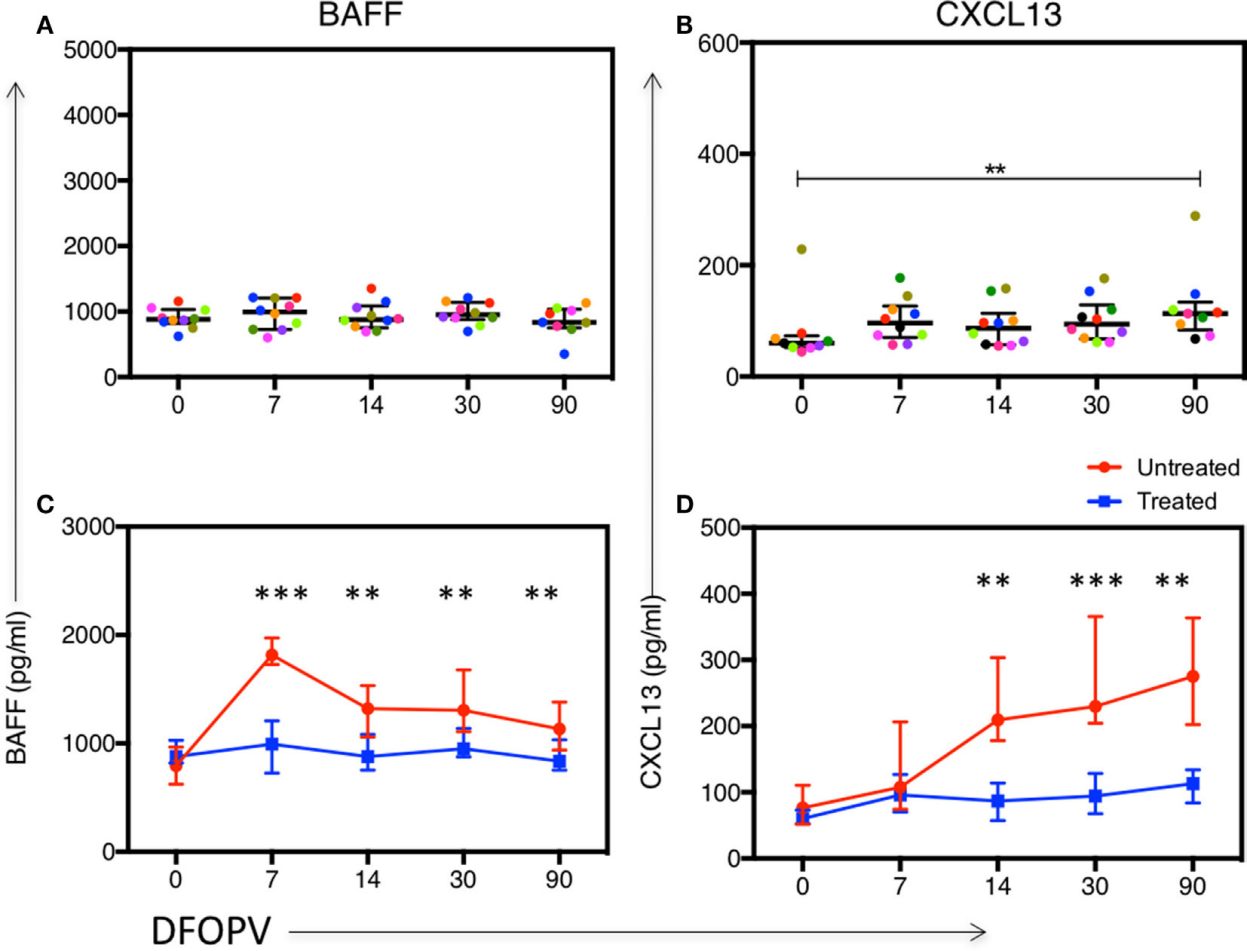
Early combination antiretroviral therapy (cART) blocks or reduces changes in plasma levels of BAFF and CXCL13. Panels **(A,B)** show kinetics of BAFF and CXCL13 levels, respectively, determined by ELISA using plasma samples obtained longitudinally from 10 women who initiated cART during Fiebig stages I–V. Panels **(C,D)** show a comparison between kinetics of BAFF and CXCL13, respectively, between untreated and early treated women. Lines represent median values. DFOPV, days following onset of plasma viremia. *p*-Values for **(A,B)** were calculated by Wilcoxon matched signed-rank test and for **(C,D)** by Mann–Whitney test (**p < 0.005, ***p < 0.0001).

Furthermore, we did not observe significant changes in median plasma BAFF levels up to 90 DFOPV (Figure 6A). However, CXCL13 levels trended toward being higher upon infection and were significantly higher at 90 DFOPV compared to baseline despite complete suppression of viral loads in most of the individuals (Figure 6B). The levels of BAFF and CXCL13 were significantly different between the untreated and early treated individuals at all time-points tested except at baseline and 7 DFOPV for CXCL13 (Figures 6C,D). Our data confirm that viremia drives the changes in B cell subset frequencies, an effect that is blocked by early treatment. Furthermore, although early cART largely abrogated the cytokine surge, there was no direct relationship between viral loads and the cytokines in untreated persons, suggesting that the early cytokine responses may be induced by infection-associated changes other than viremia.

### Emergence of Cross-Neutralizing Antibodies within 1 Year of HIV-1 Subtype C Infection

**Figure 7:**
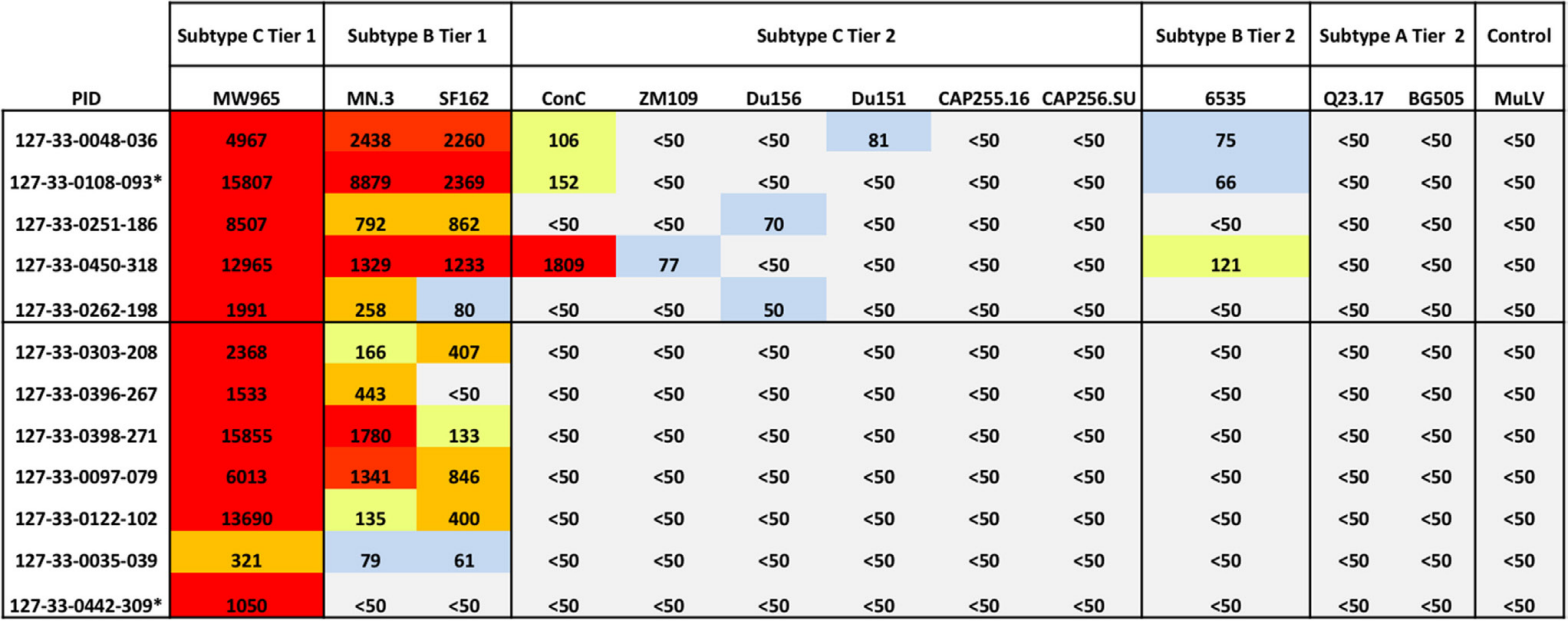
Emergence of cross-neutralization activity in plasma obtained within 1 year of HIV-1 subtype C infection. The emergence of cross-neutralization activity in plasmas from 12 patients ~1 year post detection of plasma viremia was evaluated against viruses from different clades (C, B, and A) and tiers (1 and 2) as indicated at the top. The values shown are the reciprocal dilution of plasma at which 50% of the virus was neutralized (ID50). Cases where no cross-neutralization was detected were assigned an ID50 of <1:50. ID50s are color coded for clarity; ID50 < 1:50 (gray), 1:50 to 1:100 (blue), 1:101 to 1:200 (yellow), 1:201 to 1:1,000 (orange), and >1:1,000 (red). Individuals with detectable cross-neutralization of tier 2 viruses (5/12) are grouped together. * indicates that plasma samples tested were obtained prior to 1 year of infection. MuLV was used as the negative control. Experiments were performed at least two independent times and the mean values are reported.

Given the rapid changes in frequencies of B cell subsets and increased levels of BAFF and CXCL13 observed during acute HIV-1 infection, we next determined whether the enrichment of a particular B cell subset or cytokine was associated with the emergence of cross-neutralizing antibodies, as an early predictor of cross-neutralization activity. We first probed for presence of cross-neutralizing antibodies for the 12 antiretroviral-naïve individuals using plasma collected at ~1 year PI. Antibody cross-neutralization activity was determined by standard TZM-bl assay against 12 viruses of different subtypes (C, B, and A) and tiers (1 and 2) (51) (Figure 7). As expected, we found that all individuals had detectable cross-neutralizing antibodies at 1 year PI against the tier 1 subtype C strain MW965 (100%) and most had activity against the tier 1 subtype B viruses MN.3 (92%) and SF162.LS (83%). One patient 127-33-0108-093 neutralized all three tier 1 viruses with the greatest potency at the time-point prior to initiation of cART (Figure 7). Among all subjects tested, there was weak cross-neutralization activity detected against 4/9 (44%) tier 2 viruses tested. Three patients (127-33-0048-036, 127-33-0108-093, and 127-33-0450-318) had detectable but weak cross-neutralization activity against the tier 2 subtype B viruses tested. No activity was detected against tier 2 subtype A viruses at 1 year PI (Figure 7).

To enable us to perform further analyses, individuals were categorized into those that did or did not have detectable cross-neutralization activity (regardless of the potency) against any of the tier 2 viruses (6). Using this stratification, five individuals were classified as having detectable cross-neutralization activity and seven as having no detectable cross-neutralization activity (Figure 7), and these strata were used in subsequent analysis.

### Plasma Levels of CXCL13 Early in Infection Predict Emergence of Cross-Neutralizing Antibodies 1 Year PI

We next investigated whether events occurring early upon infection could predict the emergence of cross-neutralizing antibodies 1 year PI. We found no differences between individuals with and without detectable cross-neutralization activity when comparing viral load set-point (*p* = 0.268) and contemporaneous viral loads (*p* = 0.404). Contemporaneous CD4 counts also did not distinguish between the two groups (*p* = 0.458). Notably, among individuals with detectable cross-neutralizing antibodies at 1 year, 3/5 (60%) qualified for and initiated cART due to low CD4 count within 2 years of infection compared to 2/7 (28%) of those who did not, though that relationship between emergence of cross-neutralizing antibodies and deterioration in CD4 counts was also not statistically significant (*p* = 0.558, Fisher’s exact test). One participant in the group with no detectable cross-neutralization was initiated on treatment outside of normal criteria due to pregnancy. To determine whether the expansion of a specific B cell subset following hyperacute infection was predictive of the emergence of cross-neutralizing antibodies, we compared the peak frequency of AM, TLM, and PBs and nadir levels of RM cells in the individuals with and without cross-neutralization activity and found no apparent differences in this small group of 12 individuals (data not shown).

**Figure 8:**
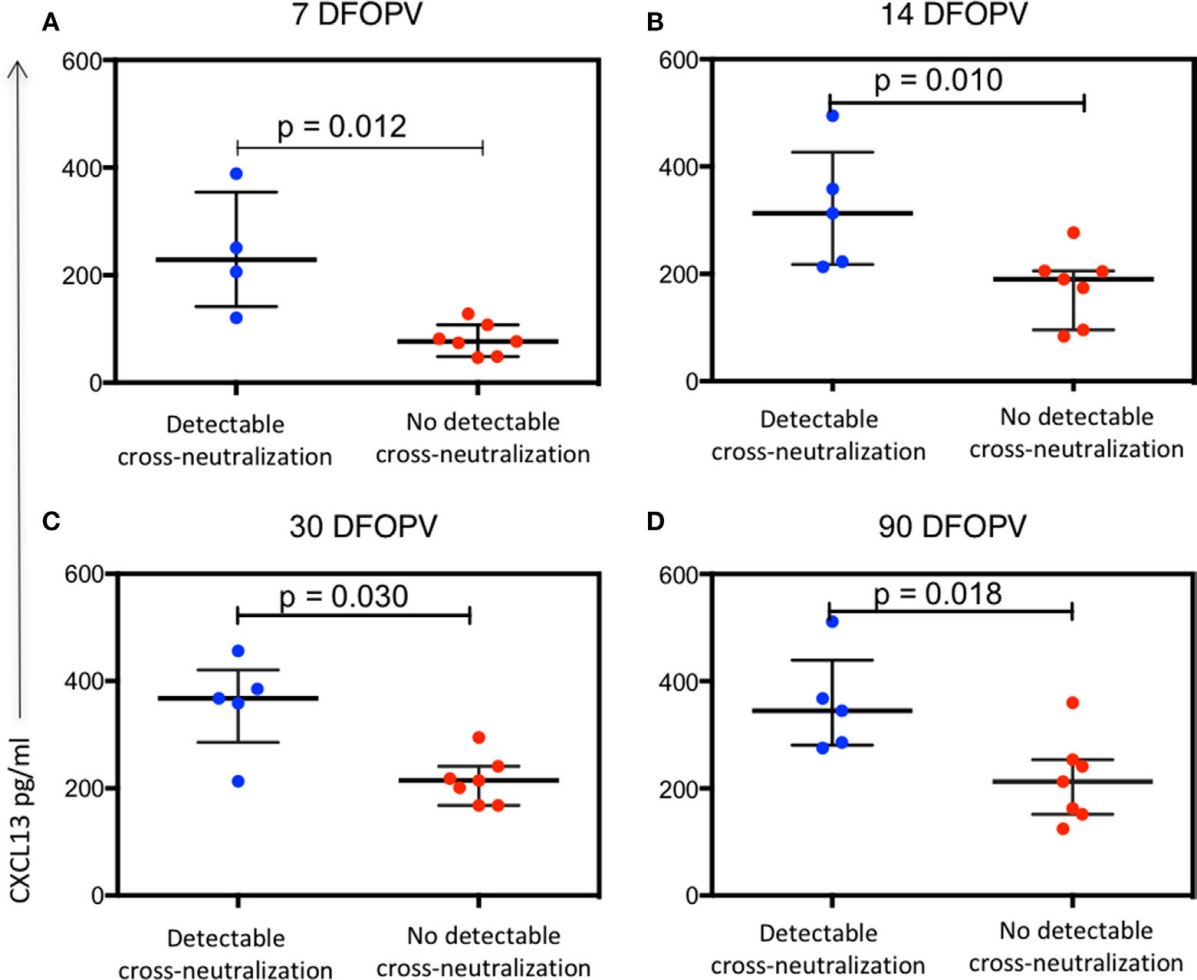
Plasma levels of CXCL13 are associated with emergence of antibody cross-neutralization activity. Panels **(A–D)** show the difference over time in plasma CXCL13 levels between individuals with detectable cross-neutralization activity (detectable cross-neutralization) and those without (no detectable cross-neutralization). *p*-Values were calculated by Mann–Whitney test. DFOPV, days following onset of plasma viremia.

Similarly, we sought to investigate whether plasma levels of CXCL13 and BAFF were associated with the emergence of cross-neutralizing antibodies. There was no significant difference between BAFF levels in the two groups at all time-points tested (data not shown). In contrast, plasma CXCL13 levels were significantly higher in those with detectable cross-neutralization activity at all early time-points tested (*p* = 0.012, 0.010, 0.030, and 0.018 for 7, 14, 30, and 90 DFOPV, respectively) (Figures 8A–D). Hence, high levels of CXCL13 early in infection were associated with emergence of cross-neutralizing antibodies within 1 year PI.

## Discussion

Development of an effective vaccine able to induce bNAbs remains a high priority for the HIV field but how these responses evolve in natural infection remains unclear. It has previously been reported that interactions between B cells and transmitted founder virus soon after infection likely shape the evolution of such antibodies (10). Thus understanding factors that influence the humoral response to HIV-1 early in natural infection could open new insights into designing an effective vaccine. We took advantage of a unique cohort in which specimens were available prior to HIV-1 infection and longitudinally during the earliest phases of infection, and determined the relationship between frequencies of B cell subsets and key B cell activating cytokines (BAFF and CXCL13) on the emergence of cross-neutralizing antibodies 1 year following infection. We show that in the absence of cART, the impact of HIV-1 infection is rapid and greatly impacts the frequencies of circulating RM, TLM, and PBs subsets, within 7 DFOPV. These frequencies rebounded although never to the baseline values by ~90 DFOPV which coincides with early stages of viral load set-point. These subset changes were associated with viral load in the regression analyses, confirming that viremia drives them. Plasma levels of BAFF and CXCL13 were also elevated in untreated people but did not show association with viral loads within that group. While BAFF levels showed a steady decrease after a peak on day 7, the levels of CXCL13 continued to rise and remained high 90 DFOPV possibly due to effects of immune activation or ongoing viral replication within lymphoid tissues. Frequencies of B cell subsets and plasma levels of BAFF did not influence emergence of cross-neutralizing antibodies. However, individuals with high plasma levels of CXCL13 early in infection were more likely to have detectable but weak cross-neutralizing antibodies at 1 year PI.

CXCL13 has previously been documented to be a biomarker of the GC activity in mice, non-human primate models, vaccine recipients and HIV-1 infected people (25–27). In those studies, participants were infected with a range of subtypes but the samples tested were mainly from primary or chronic HIV-1 infection. We report a similar observation in our cohort of 12 young African women infected with HIV-1 subtype C and in hyperacute HIV-1 infection. Importantly, pre-infection samples allowed longitudinal tracking of changes following infection, clearly demonstrating that CXCL13 is induced following infection. Similar to previous reports, viral load did not have a direct influence on the CXCL13 levels in the first 60 DFOPV. However, there was a trend toward a positive correlation by 90 DFOPV, which might be an indication of a shift toward chronic infection, a period during which CXCL13 levels and viral loads correlate positively in the absence of treatment (52–55). CXCL13 plays a crucial role in the organization of B cell follicles of secondary lymphoid organs by recruiting B cells and specific T cell subsets through its receptor CXCR5 (56, 57), thus its ability to predict emergence of cross-neutralizing antibodies is not surprising.

We report a dramatic decline in frequencies of circulating RM cells that might reflect the impact of GC destruction immediately upon establishment of HIV-1 infection (5). The mechanism by which HIV-1 results in depletion of RM cells is unclear but has significant implications for maintenance of humoral immunity. Future studies need to understand whether it is active virus replication that is responsible for RM changes or a particular viral protein, and if the latter, this would suggest potential caution in the inclusion of that protein in potential immunogens to avoid unintended detrimental immunological consequences. Of note, all the observed B cell subset changes were successfully blocked by cART initiated during Fiebig stage I-V except for an initial spike of PBs, a possible reflection of GC events where infected CD4 Tfh cells may continue to stimulate B cells within the follicles before death (58).

Despite viral loads being a good predictor of development of cross-neutralizing antibodies, which are precursors for bNAbs (6), only about 25% of individuals displaying high viral loads develop bNAbs suggesting a role for other factors. The rate of depletion of CD4 T cells has also been reported to predict the development of bNAbs (2). In our study, neither viral loads nor CD4 counts predicted the emergence of cross-neutralizing antibodies at 1 year PI. However, the independent prediction by levels of CXCL13 suggests a complex multifactorial determination of the development of cross-neutralizing antibodies. Indeed, other factors, in addition to viral loads and CD4 counts, have been reported to predict the development of cross-neutralizing activity and could have influenced the associations that we observed here. For instance, early follicular helper T cell responses, measured by the frequencies of CXCR5^+^ CD4 T cells and which we did not assess in this study, has been show to predict of the development of neutralization breadth (25, 59). In addition, the development of bNAbs has been associated with reduced control of autoreactivity (60). Importantly, the observations reported here could be limited due to the small numbers of patients available which might preclude our ability to detect associations. Furthermore, we probed for cross-neutralizing antibodies within 1 year of infection, which is very early in the development of cross-neutralizing antibodies, and certainly before the development of any bNAbs in any of the study participants. These limitations could have also reduced our ability to detect associations between B cell subsets and the emergence of cross-neutralizing antibodies.

In conclusion, acute HIV-1 subtype C infection is associated with rapid changes in B cell subsets that do not predict the emergence of cross-neutralizing antibodies within the first year of infection. Instead, our data showing an association between CXCL13 levels in acute infection and emergence of cross-neutralizing antibodies adds to growing evidence suggesting that plasma CXCL13 might be a surrogate for a functional GC compartment and serve as a biomarker to evaluate candidate vaccines for their ability to stimulate a rapid and robust GC reaction.

## Ethics Statement

This study was carried out in accordance with the recommendations of the Biomedical Research Ethics Committee of the University of KwaZulu-Natal and the Institutional Review Board of Massachusetts General Hospital with written informed consent from all subjects. All subjects gave written informed consent in accordance with the Declaration of Helsinki. The protocol was approved by the Biomedical Research Ethics Committee of the University of KwaZulu-Natal and the Institutional Review Board of Massachusetts General Hospital.

## Author Contributions

JM, A-SD, ZE, LM, BW, GA, and TN conceived the study. JM, YR, NI, AM, and KD participated in the acquisition of the data. JM, DM, and TR performed data analyses. JM drafted the manuscript with assistance from all authors. All authors gave the final approval for publication.

## Conflict of Interest Statement

The authors declare that the research was conducted in the absence of any commercial or financial relationships that could be construed as a potential conflict of interest.

## References

[1] Doria-Rose NA Klein RM Daniels MG O’Dell S Nason M Lapedes A Breadth of human immunodeficiency virus-specific neutralizing activity in sera: clustering analysis and association with clinical variables. J Virol (2010) 84(3):1631–6. 10.1128/JVI.01482-09 19923174 PMC2812355

[2] Gray ES Madiga MC Hermanus T Moore PL Wibmer CK Tumba NL The neutralization breadth of HIV-1 develops incrementally over four years and is associated with CD4+ T cell decline and high viral load during acute infection. J Virol (2011) 85(10):4828–40. 10.1128/JVI.00198-11 21389135 PMC3126191

[3] Sather DN Armann J Ching LK Mavrantoni A Sellhorn G Caldwell Z Factors associated with the development of cross-reactive neutralizing antibodies during human immunodeficiency virus type 1 infection. J Virol (2009) 83(2):757–69. 10.1128/JVI.02036-08 18987148 PMC2612355

[4] Walker LM Phogat SK Chan-Hui PY Wagner D Phung P Goss JL Broad and potent neutralizing antibodies from an African donor reveal a new HIV-1 vaccine target. Science (2009) 326(5950):285–9. 10.1126/science.1178746 19729618 PMC3335270

[5] Levesque MC Moody MA Hwang KK Marshall DJ Whitesides JF Amos JD Polyclonal B cell differentiation and loss of gastrointestinal tract germinal centers in the earliest stages of HIV-1 infection. PLoS Med (2009) 6(7):e1000107. 10.1371/journal.pmed.1000107 19582166 PMC2702159

[6] Mikell I Sather DN Kalams SA Altfeld M Alter G Stamatatos L. Characteristics of the earliest cross-neutralizing antibody response to HIV-1. PLoS Pathog (2011) 7(1):e1001251. 10.1371/journal.ppat.1001251 21249232 PMC3020924

[7] Sanchez-Merino V Fabra-Garcia A Gonzalez N Nicolas D Merino-Mansilla A Manzardo C Detection of broadly neutralizing activity within the first months of HIV-1 infection. J Virol (2016) 90(11):5231–45. 10.1128/JVI.00049-16 26984721 PMC4934761

[8] Hraber P Seaman MS Bailer RT Mascola JR Montefiori DC Korber BT. Prevalence of broadly neutralizing antibody responses during chronic HIV-1 infection. AIDS (2014) 28(2):163–9. 10.1097/QAD.0000000000000106 24361678 PMC4042313

[9] Piantadosi A Panteleeff D Blish CA Baeten JM Jaoko W McClelland RS Breadth of neutralizing antibody response to human immunodeficiency virus type 1 is affected by factors early in infection but does not influence disease progression. J Virol (2009) 83(19):10269–74. 10.1128/JVI.01149-09 19640996 PMC2748011

[10] Liao HX Lynch R Zhou T Gao F Alam SM Boyd SD Co-evolution of a broadly neutralizing HIV-1 antibody and founder virus. Nature (2013) 496(7446):469–76. 10.1038/nature12053 23552890 PMC3637846

[11] Moir S Fauci AS B cells in HIV infection and disease. Nat Rev Immunol (2009) 9(4):235–45. 10.1038/nri2524 19319142 PMC2779527

[12] Moir S Fauci AS. Insights into B cells and HIV-specific B-cell responses in HIV-infected individuals. Immunol Rev (2013) 254(1):207–24. 10.1111/imr.12067 23772622

[13] Cagigi A Nilsson A De Milito A Chiodi F. B cell immunopathology during HIV-1 infection: lessons to learn for HIV-1 vaccine design. Vaccine (2008) 26(24):3016–25. 10.1016/j.vaccine.2007.11.063 18164520

[14] Jacobson MA Khayam-Bashi H Martin JN Black D Ng V. Effect of long-term highly active antiretroviral therapy in restoring HIV-induced abnormal B-lymphocyte function. J Acquir Immune Defic Syndr (2002) 31(5):472–7. 10.1097/00126334-200212150-00003 12473834

[15] Moir S Ho J Malaspina A Wang W DiPoto AC O’Shea MA Evidence for HIV-associated B cell exhaustion in a dysfunctional memory B cell compartment in HIV-infected viremic individuals. J Exp Med (2008) 205(8):1797–805. 10.1084/jem.20072683 18625747 PMC2525604

[16] Morris L Binley JM Clas BA Bonhoeffer S Astill TP Kost R HIV-1 antigen-specific and -nonspecific B cell responses are sensitive to combination antiretroviral therapy. J Exp Med (1998) 188(2):233–45. 10.1084/jem.188.2.233 9670036 PMC2212446

[17] Notermans DW de Jong JJ Goudsmit J Bakker M Roos MT Nijholt L Potent antiretroviral therapy initiates normalization of hypergammaglobulinemia and a decline in HIV type 1-specific antibody responses. AIDS Res Hum Retroviruses (2001) 17(11):1003–8. 10.1089/088922201300343681 11485617

[18] Pensieroso S Galli L Nozza S Ruffin N Castagna A Tambussi G B-cell subset alterations and correlated factors in HIV-1 infection. AIDS (2013) 27(8):1209–17. 10.1097/QAD.0b013e32835edc47 23343911

[19] Titanji K Chiodi F Bellocco R Schepis D Osorio L Tassandin C Primary HIV-1 infection sets the stage for important B lymphocyte dysfunctions. AIDS (2005) 19(17):1947–55. 10.1097/01.aids.0000191231.54170.89 16260900

[20] Moir S Buckner CM Ho J Wang W Chen J Waldner AJ B cells in early and chronic HIV infection: evidence for preservation of immune function associated with early initiation of antiretroviral therapy. Blood (2010) 116(25):5571–9. 10.1182/blood-2010-05-285528 20837780 PMC3031405

[21] Scheid JF Mouquet H Ueberheide B Diskin R Klein F Oliveira TY Sequence and structural convergence of broad and potent HIV antibodies that mimic CD4 binding. Science (2011) 333(6049):1633–7. 10.1126/science.1207227 21764753 PMC3351836

[22] Walker LM Huber M Doores KJ Falkowska E Pejchal R Julien JP Broad neutralization coverage of HIV by multiple highly potent antibodies. Nature (2011) 477(7365):466–70. 10.1038/nature10373 21849977 PMC3393110

[23] Wu X Yang ZY Li Y Hogerkorp CM Schief WR Seaman MS Rational design of envelope identifies broadly neutralizing human monoclonal antibodies to HIV-1. Science (2010) 329(5993):856–61. 10.1126/science.1187659 20616233 PMC2965066

[24] Klein F Diskin R Scheid JF Gaebler C Mouquet H Georgiev IS Somatic mutations of the immunoglobulin framework are generally required for broad and potent HIV-1 neutralization. Cell (2013) 153(1):126–38. 10.1016/j.cell.2013.03.018 23540694 PMC3792590

[25] Cohen K Altfeld M Alter G Stamatatos L. Early preservation of CXCR5+ PD-1+ helper T cells and B cell activation predict the breadth of neutralizing antibody responses in chronic HIV-1 infection. J Virol (2014) 88(22):13310–21. 10.1128/JVI.02186-14 25210168 PMC4249103

[26] Havenar-Daughton C Lindqvist M Heit A Wu JE Reiss SM Kendric K CXCL13 is a plasma biomarker of germinal center activity. Proc Natl Acad Sci U S A (2016) 113(10):2702–7. 10.1073/pnas.1520112113 26908875 PMC4790995

[27] Dugast AS Arnold K Lofano G Moore S Hoffner M Simek M Virus-driven inflammation is associated with the development of bNAbs in spontaneous controllers of HIV. Clin Infect Dis (2017) 64(8):1098–104. 10.1093/cid/cix057 28158448 PMC5850011

[28] Liu Z Davidson A BAFF and selection of autoreactive B cells. Trends Immunol (2011) 32(8):388–94. 10.1016/j.it.2011.06.004 21752714 PMC3151317

[29] Ota M Duong BH Torkamani A Doyle CM Gavin AL Ota T Regulation of the B cell receptor repertoire and self-reactivity by BAFF. J Immunol (2010) 185(7):4128–36. 10.4049/jimmunol.1002176 20817867 PMC3263398

[30] Thien M Phan TG Gardam S Amesbury M Basten A Mackay F Excess BAFF rescues self-reactive B cells from peripheral deletion and allows them to enter forbidden follicular and marginal zone niches. Immunity (2004) 20(6):785–98. 10.1016/j.immuni.2004.05.010 15189742

[31] Dosenovic P Soldemo M Scholz JL O’Dell S Grasset EK Pelletier N BLyS-mediated modulation of naive B cell subsets impacts HIV Env-induced antibody responses. J Immunol (2012) 188(12):6018–26. 10.4049/jimmunol.1200466 22561155 PMC3370119

[32] Gupta S Clark ES Termini JM Boucher J Kanagavelu S LeBranche CC DNA vaccine molecular adjuvants SP-D-BAFF and SP-D-APRIL enhance anti-gp120 immune response and increase HIV-1 neutralizing antibody titers. J Virol (2015) 89(8):4158–69. 10.1128/JVI.02904-14 25631080 PMC4442371

[33] Ndhlovu ZM Kamya P Mewalal N Kloverpris HN Nkosi T Pretorius K Magnitude and kinetics of CD8+ T cell activation during hyperacute HIV infection impact viral set point. Immunity (2015) 43(3):591–604. 10.1016/j.immuni.2015.08.012 26362266 PMC4575777

[34] Anahtar MN Byrne EH Doherty KE Bowman BA Yamamoto HS Soumillon M Cervicovaginal bacteria are a major modulator of host inflammatory responses in the female genital tract. Immunity (2015) 42(5):965–76. 10.1016/j.immuni.2015.04.019 25992865 PMC4461369

[35] Byrne EH Anahtar MN Cohen KE Moodley A Padavattan N Ismail N Association between injectable progestin-only contraceptives and HIV acquisition and HIV target cell frequency in the female genital tract in South African women: a prospective cohort study. Lancet Infect Dis (2016) 16(4):441–8. 10.1016/S1473-3099(15)00429-6 26723758 PMC5294917

[36] Department of Health, Republic of South Africa. National Consolidated Guidelines for the Prevention of Mother-to-Child Transmission of HIV (PMTCT) and the Management of HIV in Children, Adolescents and Adults. (2014). Available from: http://www.sahivsoc.org/Files/Consolidated%20ART%20guidelines%20_Jan%202015.pdf

[37] Montefiori DC Evaluating neutralizing antibodies against HIV, SIV, SHIV in luciferase reporter gene assays. In: Coligan JE Bierer BE Margulies DH Shevach EM Strober W, editors. Current Protocols in Immunology. New York, NY: John Wiley & Sons (2004) Chapter 12: Unit 12.11. 10.1002/0471142735.im1211s64 18432938

[38] Clerici M Butto S Lukwiya M Saresella M Declich S Trabattoni D Immune activation in Africa is environmentally-driven and is associated with upregulation of CCR5. Italian-Ugandan AIDS Project. AIDS (2000) 14(14):2083–92. 10.1097/00002030-200009290-00003 11061648

[39] Howard RR Fasano CS Frey L Miller CH. Reference intervals of CD3, CD4, CD8, CD4/CD8, and absolute CD4 values in Asian and non-Asian populations. Cytometry (1996) 26(3):231–2. 10.1002/(SICI)1097-0320(19960915)26:3<231:AID-CYTO9>3.0.CO;2-H 8889397

[40] Naluyima P Eller LA Ouma BJ Kyabaggu D Kataaha P Guwatudde D Sex and urbanicity contribute to variation in lymphocyte distribution across Ugandan populations. PLoS One (2016) 11(1):e0146196. 10.1371/journal.pone.0146196 26730706 PMC4701131

[41] Kiguoya MW Mann JK Chopera D Gounder K Lee GQ Hunt PW Subtype-specific differences in Gag-protease-driven replication capacity are consistent with inter-subtype differences in HIV-1 disease progression. J Virol (2017) 91:e00253–17. 10.1128/JVI.00253-17 28424286 PMC5469260

[42] Kaleebu P French N Mahe C Yirrell D Watera C Lyagoba F Effect of human immunodeficiency virus (HIV) type 1 envelope subtypes A and D on disease progression in a large cohort of HIV-1-positive persons in Uganda. J Infect Dis (2002) 185(9):1244–50. 10.1086/340130 12001041

[43] Vasan A Renjifo B Hertzmark E Chaplin B Msamanga G Essex M Different rates of disease progression of HIV type 1 infection in Tanzania based on infecting subtype. Clin Infect Dis (2006) 42(6):843–52. 10.1086/499952 16477563

[44] Gonzalez-Garcia I Ocana E Jimenez-Gomez G Campos-Caro A Brieva JA.Immunization-induced perturbation of human blood plasma cell pool: progressive maturation, IL-6 responsiveness, and high PRDI-BF1/BLIMP1 expression are critical distinctions between antigen-specific and nonspecific plasma cells. J Immunol (2006) 176(7):4042–50. 10.4049/jimmunol.176.7.4042 16547239

[45] Odendahl M Mei H Hoyer BF Jacobi AM Hansen A Muehlinghaus G Generation of migratory antigen-specific plasma blasts and mobilization of resident plasma cells in a secondary immune response. Blood (2005) 105(4):1614–21. 10.1182/blood-2004-07-2507 15507523

[46] Wrammert J Onlamoon N Akondy RS Perng GC Polsrila K Chandele A Rapid and massive virus-specific plasmablast responses during acute dengue virus infection in humans. J Virol (2012) 86(6):2911–8. 10.1128/JVI.06075-11 22238318 PMC3302324

[47] Stacey AR Norris PJ Qin L Haygreen EA Taylor E Heitman J Induction of a striking systemic cytokine cascade prior to peak viremia in acute human immunodeficiency virus type 1 infection, in contrast to more modest and delayed responses in acute hepatitis B and C virus infections. J Virol (2009) 83(8):3719–33. 10.1128/JVI.01844-08 19176632 PMC2663284

[48] Mackay F Schneider P Rennert P Browning J. BAFF AND APRIL: a tutorial on B cell survival. Annu Rev Immunol (2003) 21:231–64. 10.1146/annurev.immunol.21.120601.141152 12427767

[49] De Milito A Nilsson A Titanji K Thorstensson R Reizenstein E Narita M Mechanisms of hypergammaglobulinemia and impaired antigen-specific humoral immunity in HIV-1 infection. Blood (2004) 103(6):2180–6. 10.1182/blood-2003-07-2375 14604962

[50] Malaspina A Moir S Kottilil S Hallahan CW Ehler LA Liu S Deleterious effect of HIV-1 plasma viremia on B cell costimulatory function. J Immunol (2003) 170(12):5965–72. 10.4049/jimmunol.170.12.5965 12794123

[51] Seaman MS Janes H Hawkins N Grandpre LE Devoy C Giri A Tiered categorization of a diverse panel of HIV-1 Env pseudoviruses for assessment of neutralizing antibodies. J Virol (2010) 84(3):1439–52. 10.1128/JVI.02108-09 19939925 PMC2812321

[52] Cagigi A Mowafi F Phuong Dang LV Tenner-Racz K Atlas A Grutzmeier S Altered expression of the receptor-ligand pair CXCR5/CXCL13 in B cells during chronic HIV-1 infection. Blood (2008) 112(12):4401–10. 10.1182/blood-2008-02-140426 18780835

[53] Cohen KW Dugast AS Alter G McElrath MJ Stamatatos L. HIV-1 single-stranded RNA induces CXCL13 secretion in human monocytes via TLR7 activation and plasmacytoid dendritic cell-derived type I IFN. J Immunol (2015) 194(6):2769–75. 10.4049/jimmunol.1400952 25667414 PMC4363079

[54] Regidor DL Detels R Breen EC Widney DP Jacobson LP Palella F Effect of highly active antiretroviral therapy on biomarkers of B-lymphocyte activation and inflammation. AIDS (2011) 25(3):303–14. 10.1097/QAD.0b013e32834273ad 21192231 PMC3322644

[55] Widney DP Breen EC Boscardin WJ Kitchen SG Alcantar JM Smith JB Serum levels of the homeostatic B cell chemokine, CXCL13, are elevated during HIV infection. J Interferon Cytokine Res (2005) 25(11):702–6. 10.1089/jir.2005.25.702 16318584

[56] Ansel KM Ngo VN Hyman PL Luther SA Forster R Sedgwick JD A chemokine-driven positive feedback loop organizes lymphoid follicles. Nature (2000) 406(6793):309–14. 10.1038/35018581 10917533

[57] Legler DF Loetscher M Roos RS Clark-Lewis I Baggiolini M Moser B. B cell-attracting chemokine 1, a human CXC chemokine expressed in lymphoid tissues, selectively attracts B lymphocytes via BLR1/CXCR5. J Exp Med (1998) 187(4):655–60. 10.1084/jem.187.4.655 9463416 PMC2212150

[58] Fukazawa Y Lum R Okoye AA Park H Matsuda K Bae JY B cell follicle sanctuary permits persistent productive simian immunodeficiency virus infection in elite controllers. Nat Med (2015) 21(2):132–9. 10.1038/nm.3781 25599132 PMC4320022

[59] Locci M Havenar-Daughton C Landais E Wu J Kroenke MA Arlehamn CL Human circulating PD-(+)1CXCR3(-)CXCR5(+) memory Tfh cells are highly functional and correlate with broadly neutralizing HIV antibody responses. Immunity (2013) 39(4):758–69. 10.1016/j.immuni.2013.08.031 24035365 PMC3996844

[60] Moody MA Pedroza-Pacheco I Vandergrift PA Chui C Lloyd KE Parks R Immune perturbations in HIV-1-infected individuals who make broadly neutralizing antibodies. Sci Immunol (2016) 1(1):aag0851 10.1126/sciimmunol.aag0851 28783677 PMC5589960

